# Inconsistent results in the analysis of *ALK* rearrangements in non-small cell lung cancer

**DOI:** 10.1186/s12885-016-2646-x

**Published:** 2016-08-05

**Authors:** Johanna S. M. Mattsson, Hans Brunnström, Verena Jabs, Karolina Edlund, Karin Jirström, Stephanie Mindus, Linnéa la Fleur, Fredrik Pontén, Mats G. Karlsson, Christina Karlsson, Hirsh Koyi, Eva Brandén, Johan Botling, Gisela Helenius, Patrick Micke, Maria A. Svensson

**Affiliations:** 1 Department of Immunology, Genetics and Pathology, Uppsala University, 751 85 Uppsala, Sweden; 2 Department of Clinical Sciences Lund, Division of Oncology and Pathology, Lund University, Lund, Sweden; 3 Department of Pathology, Regional Laboratories Region Skåne, SE-221 85 Lund, Sweden; 4 Department of Statistics, TU Dortmund University, Dortmund, Germany; 5 Leibniz Research Centre for Working Environment and Human Factors (IfADo) at Dortmund TU, Dortmund, Germany; 6 Department of Medical Sciences, Respiratory, Allergy and Sleep Research, Uppsala University, Uppsala, Sweden; 7 Department of Research and Education, Faculty of Medicine and Health, Örebro University, Örebro, Sweden; 8 School of Health Sciences, Örebro University, Örebro, Sweden; 9 Department of Respiratory Medicine, Gävle hospital, Gävle; Centre for Research and Development, Uppsala University/County Council of Gävleborg, Gävle, Sweden; 10 Department of Laboratory Medicine, Faculty of Medicine and Health, Örebro University, Örebro, Sweden; 11 Clinical Research Center, Faculty of Medicine and Health, Örebro University, Örebro, Sweden

## Abstract

**Background:**

Identification of targetable EML4-ALK fusion proteins has revolutionized the treatment of a minor subgroup of non-small cell lung cancer (NSCLC) patients. Although fluorescence in situ hybridization (FISH) is regarded as the gold standard for detection of *ALK* rearrangements, ALK immunohistochemistry (IHC) is often used as screening tool in clinical practice. In order to unbiasedly analyze the diagnostic impact of such a screening strategy, we compared ALK IHC with ALK FISH in three large representative Swedish NSCLC cohorts incorporating clinical parameters and gene expression data.

**Methods:**

*ALK* rearrangements were detected using FISH on tissue microarrays (TMAs), including tissue from 851 NSCLC patients. In parallel, ALK protein expression was detected using IHC, applying the antibody clone D5F3 with two different protocols (the FDA approved Ventana CDx assay and our in house Dako IHC protocol). Gene expression microarray data (Affymetrix) was available for 194 patients.

**Results:**

*ALK* rearrangements were detected in 1.7 % in the complete cohort and 2.0 % in the non-squamous cell carcinoma subgroup. ALK protein expression was observed in 1.8 and 1.4 % when applying the Ventana assay or the in house Dako protocol, respectively. The specificity and accuracy of IHC was high (> 98 %), while the sensitivity was between 69 % (Ventana) and 62 % (in house Dako protocol). Furthermore, only 67 % of the ALK IHC positive cases were positive with both IHC assays. Gene expression analysis revealed that 6/194 (3 %) tumors showed high ALK gene expression (≥ 6 AU) and of them only three were positive by either FISH or IHC.

**Conclusion:**

The overall frequency of *ALK* rearrangements based on FISH was lower than previously reported. The sensitivity of both IHC assays was low, and the concordance between the FISH and the IHC assays poor, questioning current strategies to screen with IHC prior to FISH or completely replace FISH by IHC.

**Electronic supplementary material:**

The online version of this article (doi:10.1186/s12885-016-2646-x) contains supplementary material, which is available to authorized users.

## Background

Lung cancer is the leading cause of death due to cancer worldwide [1]. The disease comprises histologically different entities where non-small cell lung cancer (NSCLC) presents the majority [2]. The prognosis is poor, with a five-year survival rate of approximately 15 % across all stages [3]. In recent years, comprehensive molecular studies have identified genomic aberrations leading to activating mutations in cancer drivers, prototypically presented by EGFR mutation, found in 10–50 % of adenocarcinoma patients [4, 5]. Subsequently, another cancer driver was discovered, a gene rearrangement on chromosome 2, leading to the fusion gene between echinoderm microtubule associated protein like 4 (EML4) and anaplastic lymphoma kinase (ALK) [6, 7]. This aberration is present in 3–13 % of NSCLC patients [6, 8–10].

ALK is a receptor tyrosine kinase belonging to the insulin growth factor receptor superfamily [11]. The specific physiological function of ALK is not yet clarified. However, ALK is believed to play a role in the development of the nervous system [12]. ALK deficient mice showed only mild behavioral phenotypes, proposing that ALK is not essential for viability [13–15]. In lung cancer, the fusion of *ALK* with *EML4* leads to constitutive activation of ALK, directly affecting downstream signaling and increasing cell proliferation and survival [6]. Since the discovery of the *EML4-ALK* fusion in 2007, several other *ALK* fusion partners have been described, such as kinesin family member 5B (KIF5B) [16], kinesin light chain 1 (KLC1) [17] and TRK-fused gene (TFG) [7], all fusion products leading to comparable kinase activation and transforming capacities [9, 18].

Soon after the discovery of ALK translocations in lung cancer, patients harboring this fusion gene demonstrated impressive response rates in clinical trials when treated with the ALK inhibitor crizotinib [19, 20]. The results of a subsequent phase III trial led to an accelerated approval from the United States Food and Drug Administration (FDA) of crizotinib as first-line therapy in ALK positive advanced NSCLC patients [21]. Two ALK inhibitors have been approved [22] and several are in late clinical trials [23], but the identification of the small patient subset that harbors the *ALK* rearrangement remains a diagnostic challenge.

As for many other chromosomal aberrations, fluorescence in situ hybridization (FISH) is the gold standard for the detection of *ALK* rearrangement [24]. The use of ALK inhibitors was, until recently, based on a positive FISH assay [25], although it is difficult to detect the small inversion on chromosome 2 by a fluorescence probe. Split signals can be narrow and the analysis of small biopsies, with tissue artefacts and limited amounts of cancer cells, aggravates the problem [26]. Moreover, as FISH analysis is time consuming and relatively expensive, laboratories have tried to introduce other assays to identify the rearrangement. Based on the observation that the *ALK* fusion gene results in a highly expressed fusion protein [6, 7], several immunohistochemical (IHC) assays have been established for primary screening of clinical samples, with subsequent confirmation of positive cases by FISH [24, 27–29]. The approach described above has also been discussed in several national diagnostic guidelines [30–32]. However, in 2015 the FDA approved an IHC assay (Ventana ALK (D5F3) CDx Assay, Roche Diagnostics Limited, UK) that aims to completely replace FISH for detection of *ALK* rearrangements. Evidently, both protein and genomic levels provide information to guide patient therapy, and it is surprising that gene expression, representing the molecular link between DNA and protein alterations, only rarely has been included for the assessment of *ALK* rearrangements.

The aim of this study was to evaluate the relation between *ALK* rearrangement, protein expression and gene expression in three large representative Swedish NSCLC cohorts. We compared FISH analysis with two IHC assays in 851 clinically annotated NSCLC cases. The first IHC protocol was the recently FDA approved Ventana CDx assay with the ALK clone D5F3 [33]. The second protocol applied was an in house protocol using the same antibody clone on a Dako Autostainer. The results were supplemented with gene expression data obtained from an Affymetrix microarray study.

## Methods

### Patient cohorts and clinical characteristics

The study population consisted of 851 radically resected NSCLC patients, distributed over three patient cohorts.

The first cohort (Uppsala I) included 354 patients operated 1995–2005. Frozen tissue was available for 194 patients and from these RNA was isolated and utilized for gene expression analysis, as previously described [34, 35]. Corresponding formalin-fixed paraffin-embedded (FFPE) tissue blocks were available for 188 of the 194 patients and these were included in a tissue microarray (TMA), together with 166 additional samples (*n* = 354), as previously described [36, 37].

The second cohort (Uppsala II) included 354 patients operated 2006–2010 [38, 39]. FFPE material was available from all patients, and tissue cores from each tumor block were incorporated in a TMA.

The Örebro cohort consisted of 262 patients surgically resected between 1990 and 1995 [40]. For this cohort, FFPE material was available and tissue cores from each block were included in a TMA. The TMA blocks in this cohort are constructed according to histology. Since we expected a low frequency of *ALK* rearrangements in squamous cell carcinoma, we selected TMA blocks of predominantly adenocarcinoma histology including 143 patients for further analysis.

Information on clinical parameters (age at diagnosis, sex, smoking history, tumor histology, tumor stage, performance status according to WHO) and overall survival time was obtained from the records of the population-based Uppsala-Örebro Regional Lung Cancer Register. The distribution of clinical parameters (e.g., proportion of female patients, median age, survival time, etc.) confirmed that these cohorts are representative for the operable Swedish NSCLC population [41].

### Tissue microarray construction

Tissue microarrays (TMAs) were constructed from FFPE tumor tissue. All specimens were reviewed by pathologists (Uppsala I and II (JB, HB, PM) and Örebro (MK)) and representative tumor areas were identified prior to selecting random cores from the chosen areas for TMA construction. The Uppsala TMAs were constructed using a manual tissue arrayer (MTA-1, Beecher Instruments, Sun Prairie, CA). All tumors were included in duplicates (2 × 1 mm tissue cores). The Örebro TMA consisted of tumor cores included as triplicates (3 × 1 mm), as previously described [40].

### Fluorescence in situ hybridization (FISH)

*ALK* rearrangement status was assessed by FISH using the Vysis ALK Break Apart FISH Probe Kit according to the manufacturer’s instructions. Four micron thick TMA sections were used for interphase FISH. Slides were baked for one hour at 60 °C followed by deparaffinization and rehydration. Pretreatment was performed at 80 °C for 20 min followed by protease treatment for 22 min at 37 °C. This was followed by dehydration and hybridization at 73 °C for three minutes and 37 °C overnight. Post-hybridization wash was performed at 75 °C for three minutes and then the slides were mounted with 4′,6-diamidino-2-phenylindole (DAPI) (ProLong® Gold Antifade Mountant with DAPI, ThermoFisher Scientific). Slides were analyzed under a x60-x100 oil immersion objective using an Olympus BX-61 fluorescence microscope (Center Valley, PA) equipped with filters that visualize the different wavelengths of the fluorescent probe, a charge-coupled device camera, and the CellA FISH imaging and capturing software (Olympus Soft Imaging Solution GmbH Münster, Germany). A tumor was considered *ALK* rearrangement positive if at least 15 % of 50 (minimum) or 100 analyzed tumor cells showed split probes signals or isolated orange signals in accordance with published IASLC guidelines (IASLC Atlas of ALK Testing in Lung Cancer). All FISH experiments were performed independently (by MAS) without knowledge of the IHC results for ALK protein expression. The discordant cases, which showed FISH positivity but negative IHC staining, were re-evaluated by both an independent observer (PM) and by MAS.

### Immunohistochemistry

For IHC, four micron thick FFPE sections were mounted on adhesive slides (SuperFrost Ultra Plus, Thermo Fisher Scientific, Fermont, CA) followed by incubation for 60 min at 60 °C. The IHC was performed using the same monoclonal antibody, clone D5F3, with two different protocols, from here on referred to as the “Ventana protocol” and the “Dako protocol”. As positive control a previously diagnosed *ALK* rearranged NSCLC case was used, showing strong IHC staining with both protocols.

Automated IHC with the Ventana ALK (D5F3) CDx Assay (Product no. 790–4796) was performed in a Benchmark Ultra staining module (Ventana Medical Systems, Tucson, AZ). In brief, the slides were deparaffinized using EZ prep (Product no. 950–102) followed by epitope retrieval (Cell conditioner no. 1, pH 8.5, Product no. 950–124) at 95 °C for eight minutes. After retrieval the slides were blocked for peroxidase (OptiView peroxidase inhibitor (included in Product no. 760–700)) for four minutes. IHC was performed with a monoclonal rabbit ALK antibody (Ventana, D5F3, RTU) incubated for 16 min in 36 °C. OptiView DAB IHC Detection Kit (Product no. 760–700) and OptiView Amplification Kit (Product no. 760–099) were used according to the manufacturer’s recommendations for visualization of the bound primary antibody. The slides were then counterstained with Hematoxylin II (Product no. 790–2208) for eight minutes followed by bluing reagent (Product no. 760–2037) for four minutes, prior to dehydration in graded alcohols.

The Dako protocol was based on an in house protocol with conditions optimized for the use on a Dako Autostainer. The slides were deparaffinized and pretreated in Dako PT Link (pre-treatment module) with Target Retrieval Solution, High pH (K8004, Dako, Glostrup, Denmark) at 97 °C for 20 min. Endogenous peroxidase blocking in 0.3 % hydrogen peroxide (5 min) and automated IHC was then performed on the Autostainer Link 48 (Dako) with the monoclonal rabbit ALK antibody (Cell Signaling, D5F3, Product no. 3633S) diluted 1:200 in EnVision™ FLEX Antibody Diluent (K8006, Dako) for 20 min at room temperature. Antibody incubation was followed by standard signal amplification using horseradish peroxidase (HRP) conjugated EnVision™ FLEX (K8000, Dako) at room temperature for 15 min and developed using 3,3′-diaminobenzidine (DAB) for ten minutes. The slides were counterstained with Hematoxylin (Histolab AB, Gothenburg, Sweden, 01820) for eight minutes and dehydrated in graded alcohols.

Stained slides were mounted with Pertex (Histolab AB) and scanned using the Aperio ScanScope XT (Aperio Technologies Inc, Vista, CA) whole slide scanner to generate high-resolution digital images. The scanned images were viewed in 20× magnification in the freely available software ImageScope (Aperio Technologies Inc, Vista, CA), and protein expression was manually and independently scored by two evaluators (JM and PM). The intensity of the staining was based on a four-graded scale: negative (0), weak (1), moderate (2) and strong (3). The fraction of stained tumor cells was evaluated as follows: 0 % stained cells (0), 1 % (1), 2–10 % (2), 11–20 % (3), 21–30 % (4), 31–40 % (5), 41–50 % (6), 51–75 % (7) and > 75 % (8). According to the Ventana ALK CDx Assay, the samples were classified as positive if strong (intensity 3) granular cytoplasmic brown staining was present in any percentage of tumor cells. Specimens were classified as negative if the tumor cells displayed no or only weak or moderate cytoplasmic staining.

For the Dako protocol, the percentage of stained cells was taken into account as well. A common annotation score was set for the duplicate (Uppsala cohorts) and triplicate (Örebro) tissue cores representing the same tumor sample. The ordinal scores for intensity and fraction of stained tumor cells were then multiplied to obtain values ranging between 0 and 24. This score was further dichotomized for the statistical analysis in negative (score 0–7) and positive protein expression (score 8–24).

### Gene expression microarray

RNA was extracted from frozen tumor tissue from 194 patients operated in Uppsala between 1995 and 2005 and utilized for gene expression microarray analysis on the Affymetrix HG U133 Plus 2.0 arrays (54675 probe sets, Affymetrix, Santa Clara, CA), as previously described [34, 35]. The Uppsala microarray dataset has been deposited in the Gene Expression Omnibus (GEO) data repository (GSE37745), and is openly available [35].

For ALK, two probe sets (208211_s_at; 208212_s_at) were present on the Affymetrix U133 Plus 2.0 chip set. Based on the distribution of the gene expression values, samples were dichotomized into groups with high or low expression, with a cut-off at 6.

### Statistical analysis

The Chi-squared-test was used to determine the performance of the classification. Overall survival (OS) was calculated from the date of diagnosis to the date of death. Multivariate Cox survival analysis was performed with inclusion of established prognostic parameters: age, patient performance status (not available for Örebro cohort) and stage at diagnosis. Categorization was performed as follows: age: ≤ 70 vs. > 70 years; performance status: 0 vs. I–IV, tumor stage: I vs. II–IV. Correlations between clinical parameters and gene- or protein expression values were calculated with Spearman’s rank correlation coefficient. Gene expression was used as a continuous variable and protein expression was dichotomized (high vs. low, as defined in the previous section). Adjustment for multiple testing was done by the Bonferroni–Holm method [42]. All p-values were two-sided and a statistical significance level of *p* < 0.05 was used. All analyses were performed using R version 3.2.3.

## Results

### Patient characteristics

ALK status was evaluated using TMAs from three independent NSCLC patient cohorts (Uppsala I, Uppsala II and Örebro), comprising in total 851 patients. Clinicopathological characteristics for evaluable cases are provided in Additional file 1: Table S1a-c. The distribution of clinical parameters did not differ significantly between Uppsala I and II (all comparisons *p* > 0.05). The Örebro cohort was enriched in tumors with adenocarcinoma histology, compared to Uppsala I and II, but did not differ significantly with regard to other clinical parameters. For the subset of Uppsala I patients with available fresh frozen tissue included in the analysis of gene expression using Affymetrix microarrays, the distribution of clinicopathological parameters were similar to that of the complete cohort.

### ALK status evaluated by fluorescence in-situ hybridization

ALK status using FISH was assessable for 754 (88.6 %) patients on the TMA. In non-assessable cases, either all tumor cores were missing on the TMA, the tissue present on the TMA did not contain any tumor tissue, or the hybridization was insufficient for reliable evaluation. *ALK* rearrangement was identified in 13 patients (1.7 %), including nine adenocarcinomas, two non-small cell carcinoma not otherwise specified (NOS), and two squamous cell carcinomas. Both squamous cell carcinoma cases were strongly positive for the squamous marker cytokeratin 5/6 and showed no evidence of adenosquamous differentiation. In the non-squamous cell carcinoma subgroup, comprising in total 548 patients, the frequency of ALK positivity was 2.0 % (11 patients).

### ALK status evaluated by immunohistochemistry

ALK protein expression was analyzed using automated IHC on two different platforms (Ventana Benchmark Ultra and Dako Autostainer Link 48) with two different protocols.

Using the FDA-approved Ventana ALK (D5F3) CDx Assay, ALK status was assessable for 791 patients (92.9 %). ALK positivity, defined according to Ventana CDx guidelines, was identified in 16 tumors (2.0 %), including 12 adenocarcinomas, two non-small cell carcinoma NOS, and two squamous cell carcinomas. Of the 16 ALK-positive cases, nine displayed strong homogeneous positivity in more than 75 % of tumor cells, while strong staining in 1–40 % of tumor cells was observed in the remaining seven tumors.

Using the same anti-ALK antibody clone ordered separately (Cell Signaling) together with an existing in house Dako protocol, ALK status was assessable for 806 patients (94.7 %). ALK positivity, defined based on an immunoreactivity score that takes both the intensity and the fraction of positive tumor cells into account, was identified in 12 tumors (1.5 %), including 11 adenocarcinomas and one non-small cell carcinoma NOS. Of the 12 ALK-positive tumors, three showed strong homogeneous staining in more than 75 % of the tumor cells, while lower immunoreactivity scores and a varying pattern with regard to staining intensity and fraction of positive tumor cells was observed for the remaining nine tumors.

### Re-evaluation of TMA results on whole tissue sections

**Fig. 1:**
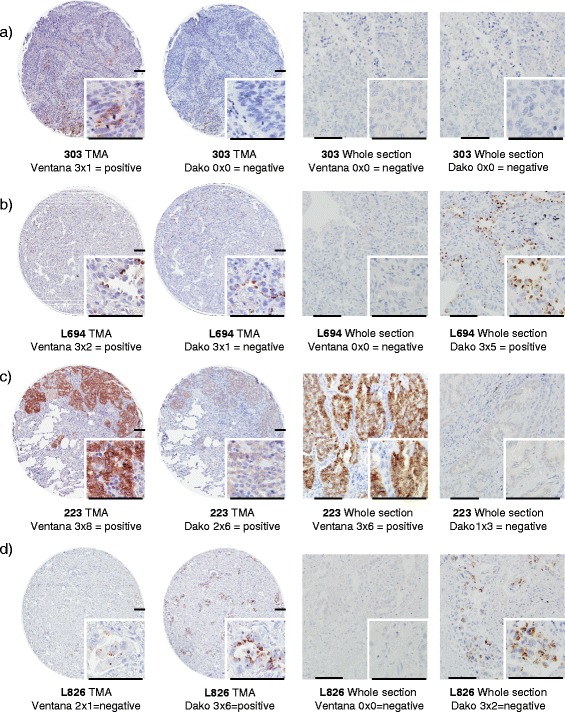
Re-evaluation of the discordant cases: **a** 303: Using the Ventana IHC protocol the staining of the tissue core was annotated as positive (strong staining in 1 % of the tumors cells). The corresponding whole section did not show any protein expression. The Dako protocol was negative on both the TMA and the whole section. The FISH analysis was also negative. **b** L694: Using the Ventana IHC protocol the staining of the tissue core was annotated as positive (strong staining in 2–10 % of tumors cells). The corresponding whole section did not show any protein expression. The Dako protocol resulted in an opposite assessment with a negative result on the TMA and a positive result on the whole section. The FISH analysis was negative for this case. **c** 223: This case was annotated as positive with the Dako protocol on the TMA (moderate staining in 41–50 % of tumor cells) but was scored negative when the whole section was evaluated. This case was defined as positive both with Ventana IHC assay and the FISH assay. **d** L826: This case was annotated as positive with the in house Dako protocol on the TMA (strong staining in 41–50 % of tumor cells) but was scored negative when the whole section was evaluated. The Ventana IHC assay and the FISH analysis were negative on the TMA as well as on the whole sections. The scale bar in all images represents 100 μm

Although TMAs are excellent for screening, the selected tumor cores from each case do not necessarily represent the whole tumor and thus results may be influenced by intratumoral heterogeneity, a scenario that mimics small diagnostic biopsies in the clinical setting. To address this potential issue, all tumors that showed ALK-positivity in at least one assay, or a contradicting result in one or two other assays (24 tumors), were re-evaluated on a corresponding whole tissue section using all three assays (FISH and the two IHC protocols). All cases interpreted as FISH positive on the TMA were also annotated as positive in the FISH analysis on whole tissue section. The re-evaluation of the Ventana IHC analysis led to the re-annotation of two cases from positive to negative (303 and L694) (Fig. 1a and b). The re-evaluation of the Dako IHC analysis resulted in the re-classification of two cases from positive to negative (223 and L826) and one case from negative to positive (L694) (Fig. 1b-d). For these five cases we used the results based on the whole tissue sections in the further analysis. Final results and percentages after re-evaluation are seen in Table 1.

**Table 1:**
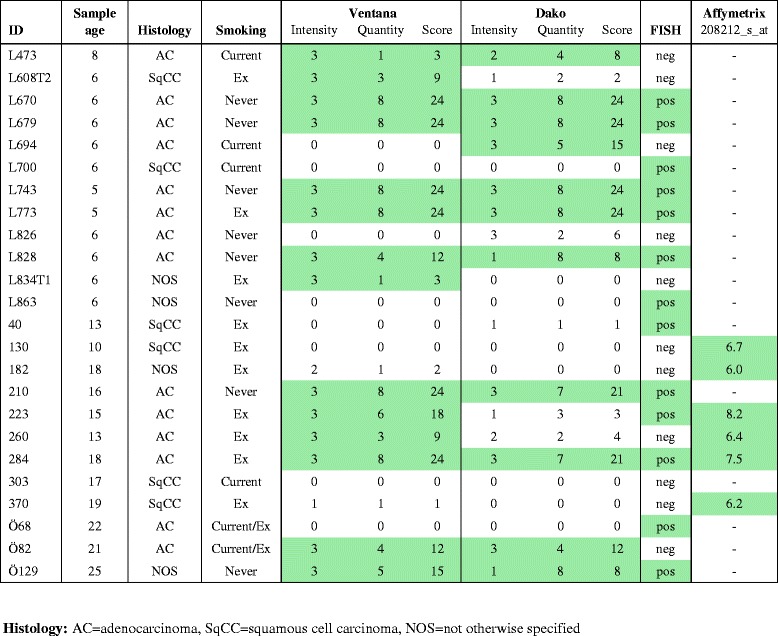
Results of all cases with ALK positivity in at least one of the assays. All cases that demonstrated positivity in one of the assays (Ventana protocol, Dako protocol, FISH analysis or Affymetrix gene expression microarray) were re-analyzed on whole tissue sections and the results are given in the table. Defined positivity is indicated by green color. Included in the table are also information about the age of the sample (years), histology and smoking

After re-evaluation on whole tissue section, the number of FISH positive cases remained unchanged (1.7 %). The number of positive cases declined to 14 (1.8 %) cases and to 11 (1.4 %) cases with the Ventana assay and the Dako assay, respectively.

### Comparison between the two immunohistochemical protocols

First, we wanted to evaluate the agreement between the two IHC assays (both using anti-ALK clone D5F3), a comparison which is important as the Ventana CDx Assay recently received FDA-approval to replace FISH analysis for the selection of patients eligible for ALK inhibitor treatment and is likely to replace existing in house validated protocol.

**Fig. 2:**
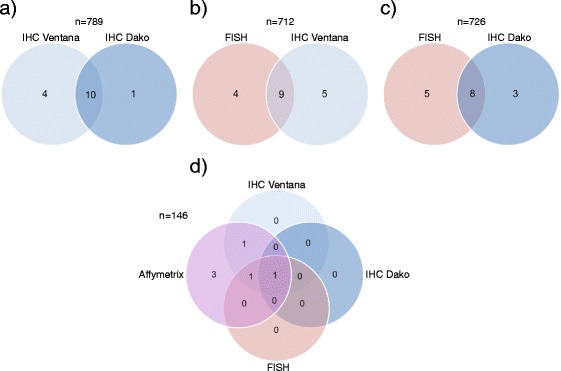
Venn diagram of ALK positivity based on different analyses. **a** Immunohistochemical positivity was compared between the Ventana IHC and Dako protocol. **b** Samples with positive FISH were compared to samples with positive Ventana protein expression. **c** Samples with positive FISH were compared to samples with positive protein expression when using the Dako protocol. **d** Samples with positive FISH, Ventana IHC, in house Dako IHC or Affymetrix gene expression were compared to each other

**Fig. 3:**
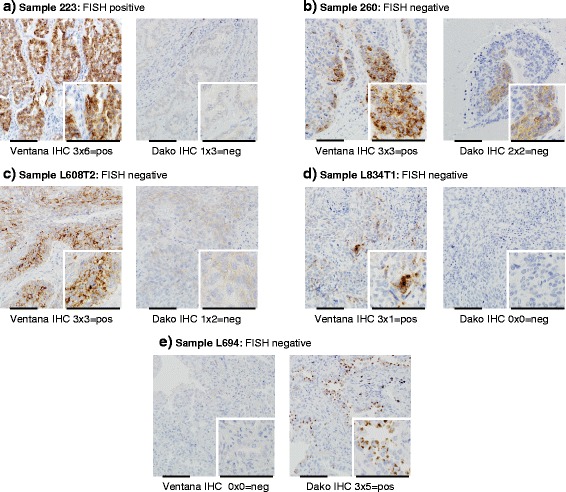
Discordant cases with the immunohistochemical assays: **a** 223: The case was defined as positive with the Ventana protocol but did not show relevant staining with the Dako protocol. The corresponding FISH analysis indicated an *ALK* rearrangement (cores and whole section). **b** 260: This case revealed a positive staining with the Ventana assay but was not positive according the defined cut-off of the in house Dako assay. In this case the FISH analysis did not demonstrate a rearrangement. **c** L608T2: This case demonstrated a positive staining with the Ventana assay, but was negative according to the Dako assay. This case was negative with FISH. **d** 2L834T1: This sample was positive with the Ventana assay, but negative with the Dako assay. The FISH analysis did not show a rearrangement. **e** L694: This case was negative according to the Ventana staining, but positive according to the Dako assay. FISH did not demonstrate a rearrangement. The scale bar in all images represents 100 μm

Altogether 789 cases were evaluable for both IHC assays. Of 15 cases that were positive in at least one of the IHC assays, only ten (66.7 %) were positive with both protocols (Fig. 2a). The Ventana protocol defined four cases as positive (strong staining in 1–50 % of the cancer cells) but these were annotated as negative with the Dako protocol (weak to moderate staining in 1–30 % of the cells, below our defined cut-off). One of the four cases annotated as positive with the Ventana protocol was of squamous cell histology (Fig. 3a-d). The Dako protocol defined one additional case as positive, with strong staining in 31–40 % of tumor cells (Fig. 3e). This case was completely negative with the Ventana protocol. Thus, the ‘retrospectively screening’ for ALK inhibitor therapy eligibility, using the Ventana CDx Assay instead of our in house protocol, led to the identification of four additional patients who else would not have been further considered for targeted treatment.

### Comparison between FISH and IHC Ventana CDx protocol

The concordance between ALK status determined by FISH and IHC is of high importance, when IHC is suggested to replace FISH for identification of the patient subset likely to benefit from ALK inhibitor therapy.

**Fig. 4:**
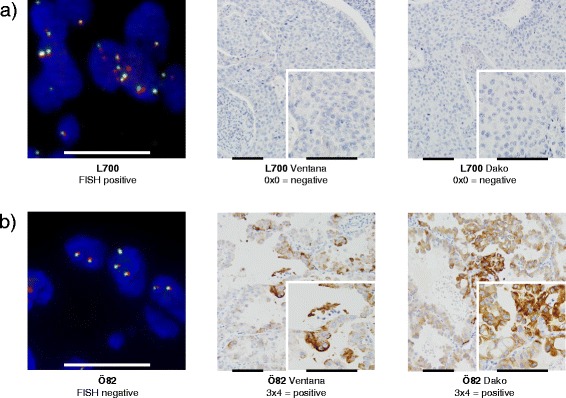
Discordant case between FISH and immunohistochemical Ventana assay: **a** L700: The FISH assay indicated an *ALK* fusion gene, but immunohistochemical staining using the Ventana protocol demonstrated no relevant protein expression. **b** Ö82: This case was negative with the FISH assay, but showed a clear positive staining with the Ventana and the in house assay. The scale bar in the FISH and IHC images represents 10 and 100 μm, respectively

In our study, 712 tumors could be evaluated with both FISH and IHC using the Ventana CDx Assay. Of 13 FISH positive tumors, nine were found to be positive according to the Ventana CDx Assay (Fig. 2b). These nine concordant cases showed strong protein expression in 21–100 % of the tumor cells. Additionally, four cases were rearranged according to FISH but did not display positivity on the protein level (Fig. 4a). Furthermore, five tumors were ALK positive according to the Ventana CDx Assay, but were not found to be rearranged using FISH (Fig. 4b). These five cases displayed strong staining in between 1 and 30 % of the cells. Considering FISH as the reference method, the sensitivity of the Ventana CDx Assay was 69.2 % with a specificity of 99.3 % and an accuracy of 99 % (Fig. 2b).

### Comparison between FISH and IHC Dako protocol

Next, we assessed the overlap between FISH and IHC positive tumors using the Dako IHC-protocol. The number of assessable tumors evaluated with both assays was 726. Of the 13 FISH positive tumors, eight were also positive according to the Dako protocol (Fig. 2c). Six of these eight concordant cases showed strong positive protein expression ranging between 50 and 100 % in the cells (scores ranging between 15 and 24), and two cases displayed weak staining in > 75 % of the cells (score 8). Additionally, five cases were rearranged according to FISH analysis, but these cases showed negative protein expression (Fig. 4a). Furthermore, according to the IHC analysis, three additional cases displayed positive protein expression when using the Dako protocol but were FISH negative (Fig. 4b). Considering FISH as the reference method the sensitivity of the in house IHC assay was 61.5 % with a specificity of 99.6 % and an accuracy of 98.9 % (Fig. 2c).

**Fig. 5:**
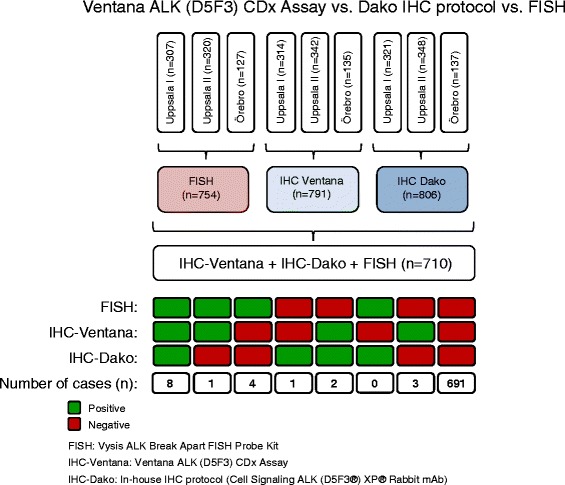
Comparisons between the FISH assay and the both IHC assays. Summary of the cases from the three cohorts available for ALK evaluation with FISH, the Ventana assay and the Dako assay

The results of the comparisons between FISH and both IHC assays are illustrated in Fig. 5.

### Gene expression microarray

Today, it is unclear if genomic rearrangement leading to the expression of the *ALK* fusion gene is the sole predictor of response to ALK inhibitor therapy or if patients that show high ALK levels, regardless of the underlying cause, are equally receptive to therapeutic ALK inhibition. To evaluate the pattern of high ALK expression we compared mRNA levels to protein expression and FISH status in a subset of patients.

**Fig. 6:**
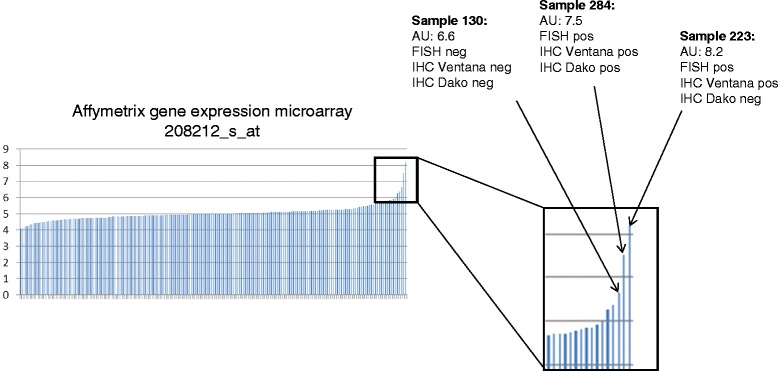
Gene expression of ALK using microarray analysis. Affymetrix gene expression data of probe set 208212_s_at from 194 NSCLC cases from the Uppsala cohort I. Relative gene expression signals are given as log-values. Samples with positivity in one of the FISH or IHC assays are designated in the magnification. All other cases with available gene expression data were negative

Fresh frozen tissue of 194 cases from the Uppsala I cohort (54.8 %) were analyzed by Affymetrix gene expression microarray for ALK gene expression represented by two probe sets. With the probe set 208211_s_at, two cases (1.0 %) were positive (gene expression values of ≥ 6), while with probe set 208212_s_at, six cases (3.1 %) were positive, with an overlap of two cases with probe set 208211_s_at. Thus, six cases were defined as gene expression positive (Fig. 6). Only three of the six cases were positive according to one of the three other ALK assays (FISH, Ventana and Dako IHC) (Fig. 2d). Importantly, the two cases with the highest gene expression (gene expression values 8.2 and 7) values were also FISH positive. Three of the six cases were of the adenocarcinoma histology, one was NSCLC NOS, while the two remaining cases were squamous cell carcinomas.

### Correlation to clinical parameters

Clinical parameters, including survival, were available for all three cohorts as listed in Additional file 1: Table S1. As expected, the correlation analysis revealed that ALK positivity was associated with lower age, female sex, and non-smoking (Table 2), but the grade of association was variable depending on the assay used, with significant association only for smoking status and gender. The ALK positive samples with the Dako protocol showed the strongest associations to age (*p* = 0.11), gender (*p* = 0.03) and smoking status (*p* < 0.001), while ALK positivity in the Ventana assay and in the FISH assay demonstrated clearly weaker associations except for smoking (*p* < 0.001).

**Table 2:** Correlations between clinical parameters and the three ALK rearrangement detection methods in all three cohorts

	FISH		Ventana IHC		Dako IHC	
Variable	Correlation	*P*-value	Correlation	*P*-value	Correlation	*P*-value
Gender	0.020	0.601	0.043	0.230	0.078	0.028
Age	−0.002	0.959	−0.049	0.167	−0.057	0.108
Stage	0.047	0.197	0.035	0.326	0.042	0.238
Smoking	−0.188	<0.001	−0.140	<0.001	−0.166	<0.001

*FISH* positive vs negative, *Ventana IHC Assay* intensity 3 vs the other intensities, *Dako IHC Assay* score <8 vs. ≥8, *Age at diagnosis* ≤70 years vs. >70 years, *Stage* IA + IB vs. II-IV, *Smoking* smokers + ex-smokers vs. never smokers

A Cox regression model was applied to test the association of ALK status with survival combining all three cohorts. A weak tendency towards a better overall survival for ALK positive patients was seen with all three assays when all histologies were analyzed together (*p* = 0.26–0.37, all analyses, Table 3).

**Table 3:** Cox regression analysis of ALK positivity in the Uppsala I cohort, the Uppsala II cohort and the Örebro cohort when combining all histologies

	HR^a^	Univariate 95 % CI^b^	*p*-value	Adj. *p*-value^c^	HR^a^	Multivariate 95 % CI^b^	*p*-value	Adj. *p*-value^c^
NSCLC								
ALK (FISH)	0.668	0.30–1.47	0.308	1	0.628	0.26–1.53	0.307	1
ALK (Ventana)	0.649	0.29–1.45	0.292	1	0.664	0.27–1.62	0.369	1
ALK (Dako)	0.567	0.21–1.52	0.258	0.775	0.567	0.18–1.79	0.333	0.775

^a^HR = Hazard Ratio

^b^95% CI = Confidence Interval

^c^Adj. *p*-value = Bonferroni-Holm

## Discussion

In this study we evaluated the frequency of *ALK* rearrangements as well as the relation between *ALK* fusion, protein expression and gene expression in three representative Swedish NSCLC cohorts. We found that the incidence of *ALK* fusions in our NSCLC population is lower than previously reported, independent of the assay used. Also, in contrast to previous studies [43–46], the different assays did not show convincing concordance, indicating that they are not interchangeable. Thus, in the clinical setting they should be used with caution. Notably, using FISH we found *ALK* rearrangements in squamous cell carcinomas (2 of 13 cases, 15 %), a clinically relevant finding that deserves specific attention, as 15 % of positive cases would not have been identified if the analysis had been focused on non-squamous cancers only.

Previous reports mostly demonstrated *ALK* rearrangement frequencies of 3-5 % in unselected patient populations [47–49], 3–25 % in adenocarcinomas [50–53], and 33 % in highly selected patient populations (EGFR wild-type, female, non/light smokers) [8]. Our frequency is lower, with 1.7 % FISH positive cases, and 2.0 % positive cases in non-squamous patients. Several factors might have influenced this result: (1) this study is performed on a Swedish population and maybe these patients are less prone to develop *ALK* fusion genes. One other study from Finland [54] evaluated 87 NSCLC patients enriched for non-smokers and adenocarcinomas, and displayed an *ALK* fusion frequency of 5.7 %, which would also be considered as comparably low for selected patients. (2) The frequency of *ALK* fusion in resected, hence localized tumors, is maybe lower than in advanced patients that were mostly analyzed in other studies. Indeed, one large study of resected adenocarcinoma patients, revealed a frequency of only 2.2 % FISH positive cases [28], thus only slightly higher than in in our study. (3) Based on these arguments, we believe that the frequency of around 2 % reflects the real clinical scenario in this unselected Swedish population of localized NSCLC.

A clinically relevant finding is the imperfect overlap of IHC and FISH. The sensitivity of the Ventana protocol and the Dako protocol was only 69.2 and 61.5 %, respectively, when FISH results were considered as the gold standard. Although, the specificity was high, 99.3 and 99.6 % respectively, the performance of the assays, detecting around two thirds of positive cases, might be regarded as insufficient. The low sensitivity witnessed in this study was in contrast to other studies demonstrating higher sensitivities ranging between 90 and 100 % for the antibody clone D5F3 [45, 55–63], suggesting that IHC as a screening method may be a complement to, or completely replace ALK-FISH [30, 31, 64]. A problem in several of these studies is that tissue specimens were screened with IHC and later confirmed with FISH providing an excellent but misleading high sensitivity [28, 65–67]. Indeed, there are recent studies comparing FISH and IHC techniques with sensitivities below 90 % [47, 68–70]. Also the analysis of the two clinical phase III studies 1014 and 1029 leading to the FDA approval of the Ventana ALK (D5F3) CDx Assay demonstrated a sensitivity of 86 and 93 %, respectively, i.e., leading to the exclusion of 14 % patients from a highly effective therapy option in study 1014 ([71], NCT01639001). These results should be considered when institutions incorporate ALK-IHC as an initial screening method prior to FISH testing, a strategy that is already discussed in most of the current guidelines.

The comparison of our previously used in house Dako IHC protocol with the FDA approved Ventana assay revealed a surprising discordance, although both protocols use the same antibody clone D5F3. Some of the inconsistencies may be explained by analytical factors such as different staining instruments and different secondary signal amplifications methods. Moreover, we used a different cut-off defining positivity for the in house assay. Our study indicates a higher sensitivity when using the Ventana system compared to the in house protocol (69 % vs. 62 %). Together with the FDA approved standardization and the straight forward annotation of positivity, the use of the Ventana protocol will be more appropriate in the diagnostic setting.

Another question that emerged, considering the surprisingly high discrepancy between IHC and FISH, is if FISH should be regarded as the gold standard in the clinical setting? Although FISH has many favorable features, for instance being able to detect *ALK* rearrangements regardless of fusion partner, several arguments contrast this assumption: FISH evaluation is dependent on many pre-analytical factors, such as fixation time and what type of fixative used. Another factor affecting the result is the storage condition of the blocks and the cut sections. Analytical factors such as the hybridization process, although standardized for most protocol, may not work optimally for every sample, since it is dependent on how the tissue is composed [72]. Also, the read out with a split event in a small part of the short arm of chromosome 2 is difficult to be surely detectable and the cut off of 15 % of split positive cells is maybe not optimal. Thus, the interpretation of ALK status in the routine settings presents the highest challenge in diagnostics, in particular on small biopsy cores and when the hybridization of the probes is suboptimal. With this background it would be reasonable in clinical diagnostics to test with IHC and FISH in parallel. A more pragmatic alternative would be to screen with IHC and to test all patients with a higher probability (non-smokers, younger age) of *ALK* rearrangements with FISH. It should be stressed that also histological features, as the recently described mucinous-cribriform/papillary histologic growth pattern, are strongly correlated with the presence of fusion genes [73, 74] and thus can be used as a criteria for ALK-testing.

To circumvent these problems, other techniques have been introduced to detect *ALK* rearrangement in the diagnostic setting. The group of Nitta et al. developed a IHC detection system with increased sensitivity that is used in combination with a bright field in situ hybridization assay, providing a co-visualization of protein expression and genomic ALK rearrangement [75].

Other alternatives for ALK fusion gene detection includes RT-PCR based assays [76], but these techniques are relying on the knowledge of the fusion transcript variants probably resulting in false negative cases. Recently, next generation sequencing methods have been developed, like RNA sequencing, to catch hitherto unknown fusion genes [77]. The nCounter system (NanoString Technologies, Seattle, WA) presents a new promising method to quantify RNA without using RT-PCR. Multiple RNA molecules were directly quantified after simultaneous hybridization [78]. For ALK analysis NanoString uses a multiplex combination of *ALK* fusion transcripts detection and determination of an ALK 3′ transcript overexpression. This is a relatively inexpensive and fast technique that has showed highly concordant results with FISH and IHC [79]. Furthermore, the simultaneous detection of other NSCLC fusion genes (RET, ROS1, NRG1, and BRAF) and the MET skipping transcript is possible [80]. We believe that this technology will replace both FISH and IHC analyses in the future diagnostic algorithms.

Based on the notion that protein expression correlates with *ALK* rearrangement, we evaluated how gene expression levels are associated with the ALK status. We found that the two cases with the highest gene expression demonstrated FISH and IHC positivity. Although higher gene expression of the 3′ end of ALK has been used as one criterion in recently presented detection methods [79], the significance of a general higher ALK expression without fusion has not been evaluated. It is to speculate, if the four FISH negative cases with high gene expression observed in our study present a subgroup of patients that may also benefit from ALK inhibitor therapy. The same situation could refer to FISH negative cases with high protein expression, in our study altogether six patients. Indeed, there are several case studies reporting objective responses to crizotinib therapy in FISH negative cases [49, 81, 82].

The major strength of our study is the unbiased strategy to perform all analysis independently and in parallel in this large NSCLC population. Indeed, with more than 700 patients, our study presents one of the largest systematic analyses of ALK status in NSCLC patients. The use of TMAs could be considered as a disadvantage; however, from the clinical perspective the TMA with small tissue cores reflects the small sample size of biopsies tested in routine diagnostics. While none of the FISH results changed when compared to the analysis of the corresponding whole section, the IHC on whole section led to re-annotation of two of 24 cases for the Ventana assay and three of 24 of the in house assay. This difference most likely indicates tissue heterogeneity of protein expression, leading to the described uncertainty of IHC in small biopsies. Naturally, a comparative analysis of TMA cores and whole section would be of interest, but was beyond the scope of this study.

## Conclusions

The results of our large comparative study indicate a low frequency of ALK aberrations in the Swedish population of NSCLC patients. Although we tested a FDA approved IHC assay to detect ALK protein expression, the sensitivity is relatively low, thus questioning its suitability for use as a screening assay. The discordant results also stress the need for careful validation of all ALK detection methods before they can be implemented into clinical practice. Finally, we demonstrated that there are patients with high ALK protein and mRNA levels that do not harbor *ALK* rearrangement according to FISH, maybe indicating a distinct group of patients that would also benefit from ALK inhibition.

## Abbreviations

ALK, anaplastic lymphoma kinase; DAB, 3,3′-diaminobenzidine; DAPI, 4′,6-diamidino-2-phenylindole; EML4, echinoderm microtubule associated protein like 4; FDA, United States Food and Drug Administration; FFPE, formalin-fixed paraffin-embedded; FISH, fluorescence in situ hybridization; HRP, horseradish peroxidase; IASLC, International Association for the Study of Lung Cancer; IHC, immunohistochemistry; KIF5B, kinesin family member 5B; KLC1, kinesin light chain 1; NOS, not otherwise specified; NSCLC, non-small cell lung cancer; OS, overall survival; TFG, TRK-fused gene; TMA, tissue microarray

## Additional file

Additional file 1: a) Patient characteristics of NSCLC patients in the Uppsala I cohort (surgically resected between 1995 and 2005). b) Patient characteristics of NSCLC patients in the Uppsala II cohort (surgically resected between 2006 and 2010). c) Patient characteristics of NSCLC patients in the Örebro cohort (operated between 1990 and 1995). (DOC 97 kb)
