# Supplementary material for: Inconsistent results in the analysis of ALK rearrangements in non-small cell lung cancer
Source: BMC Cancer. 2016 Aug 5;16:603. doi: 10.1186/s12885-016-2646-x (PMC4974795; doi:10.1186/s12885-016-2646-x)
Supplement: Additional file 1: — a) Patient characteristics of NSCLC patients in the Uppsala I cohort (surgically resected between 1995 and 2005). b) Patient characteristics of NSCLC patients in the Uppsala II cohort (surgically resected between 2006 and 2010). c) Patient characteristics of NSCLC patients in the Örebro cohort (operated between 1990 and 1995). (DOC 97 kb) [file 12885_2016_2646_MOESM1_ESM.doc]

**Supplemental table 1.**

**a)** Patient characteristics of NSCLC patients in the Uppsala I cohort (surgically resected between 1995-2005).

|  | **Uppsala I**  **Gene expression, n (%)** | **Uppsala I**  **FISH, n (%)** | **Uppsala I**  **Ventana, n (%)** | **Uppsala I**  **Dako, n (%)** |
| --- | --- | --- | --- | --- |
| **All cases** | 194 (100) | 307 (100) | 314 (100) | 321 (100) |
| **Sex** |  |  |  |  |
| Male | 105 (54.1) | 164 (53.4) | 170 (54.1) | 172 (53.6) |
| Female | 89 (45.9) | 143 (46.6) | 144 (45.9) | 149 (46.4) |
| **Age at diagnosis** |  |  |  |  |
| ≤70 | 150 (77.3) | 214 (69.7) | 218 (69.4) | 221 (68.8) |
| >70 | 44 (22.7) | 93 (30.3) | 96 (30.6) | 100 (31.2) |
| **Smoking History** |  |  |  |  |
| Current + ex smoker | 179 (92.3) | 279 (90.9) | 287 (91.4) | 292 (90.9) |
| Never smoker | 15 (7.7) | 26 (8.5) | 25 (8.0) | 27 (8.4) |
| Missing data | 0 | 2 (0.7) | 2 (0.6) | 2 (0.6) |
| **TNM-stage at diagnosis** |  |  |  |  |
| IA | 38 (19.6) | 72 (23.5) | 75 (23.9) | 76 (23.7) |
| IB | 90 (46.4) | 134 (43.6) | 133 (42.4) | 137 (42.7) |
| IIA | 6 (3.1) | 10 (3.3) | 10 (3.2) | 10 (3.1) |
| IIB | 29 (14.9) | 38 (12.4) | 42 (13.4) | 43 (13.4) |
| IIIA | 21 (10.8) | 30 (9.8) | 31 (9.9) | 33 (10.3) |
| IIIB | 6 (3.1) | 15 (4.9) | 14 (4.5) | 13 (4.0) |
| IV | 4 (2.1) | 8 (2.6) | 9 (2.9) | 9 (2.8) |
| **Histology** |  |  |  |  |
| Adenocarcinoma | 106 (54.6) | 171 (55.7) | 171 (54.5) | 175 (54.5) |
| Squamous cell carcinoma | 63 (32.5) | 100 (32.6) | 109 (34.7) | 110 (34.3) |
| NOS | 25 (12.9) | 36 (11.7) | 34 (10.8) | 36 (11.2) |
| **WHO performance status** |  |  |  |  |
| 0 | 105 (54.1) | 164 (53.4) | 166 (52.9) | 167 (52.0) |
| 1 | 73 (37.6) | 113 (36.8) | 116 (36.9) | 122 (38.0) |
| 2 | 12 (6.2) | 25 (8.1) | 26 (8.3) | 26 (8.1) |
| 3 | 4 (2.1) | 4 (1.3) | 5 (1.6) | 5 (1.6) |
| 4 | 0 | 1 (0.3) | 1 (0.3) | 1 (0.3) |
| **Mean follow-up (months)** | 58.4 | 59.2 | 59.0 | 58.5 |

FISH=fluorescence in situ hybridization

NOS=not otherwise specified

**b)** Patient characteristics of NSCLC patients in the Uppsala II cohort (surgically resected between 2006-2010).

|  | **Uppsala II**  **FISH, n (%)** | **Uppsala II**  **Ventana, n (%)** | **Uppsala II**  **Dako, n (%)** |
| --- | --- | --- | --- |
| **All cases** | 320 (100) | 342 (100) | 348 (100) |
| **Sex** |  |  |  |
| Male | 153 (47.8) | 170 (49.7) | 171 (49.1) |
| Female | 167 (52.2) | 172 (50.3) | 177 (50.9) |
| **Age at diagnosis** |  |  |  |
| ≤70 | 208 (65.0) | 224 (65.5) | 228 (65.5) |
| >70 | 112 (35.0) | 118 (34.5) | 120 (34.5) |
| **Smoking History** |  |  |  |
| Current + ex smoker | 285 (89.1) | 302 (88.3) | 307 (88.2) |
| Never smoker | 35 (10.9) | 40 (11.7) | 41 (11.8) |
| **TNM-stage at diagnosis** |  |  |  |
| IA | 134 (41.9) | 140 (40.9) | 143 (41.1) |
| IB | 67 (20.9) | 76 (22.2) | 76 (21.8) |
| IIA | 39 (12.2) | 40 (11.7) | 40 (11.5) |
| IIB | 31 (9.7) | 33 (9.6) | 33 (9.5) |
| IIIA | 43 (13.4) | 46 (13.5) | 49 (14.1) |
| IIIB | 0 (0.0) | 0 (0.0) | 0 (0.0) |
| IV | 6 (1.9) | 7 (2.0) | 7 (2.0) |
| **Histology** |  |  |  |
| Adenocarcinoma | 189 (59.1) | 200 (58.5) | 205 (58.9) |
| Squamous cell carcinoma | 92 (28.8) | 102 (29.8) | 103 (29.6) |
| NOS | 39 (12.2) | 40 (11.7) | 40 (11.5) |
| **WHO performance status** |  |  |  |
| 0 | 198 (61.9) | 206 (60.2) | 209 (60.1) |
| 1 | 119 (37.2) | 133 (38.9) | 136 (39.1) |
| 2 | 3 (0.9) | 3 (0.9) | 3 (0.9) |
| 3 | 0 (0.0) | 0 (0.0) | 0 (0.0) |
| 4 | 0 (0.0) | 0 (0.0) | 0 (0.0) |
| **Mean follow-up (months)** | 50.1 | 50.2 | 49.9 |

FISH=fluorescence in situ hybridization

NOS=not otherwise specified

**c)** Patient characteristics of NSCLC patients in the Örebro cohort (operated between 1990-1995).

|  | **Örebro**  **FISH, n (%)** | **Örebro**  **Ventana, n (%)** | **Örebro**  **Dako, n (%)** |
| --- | --- | --- | --- |
| **All cases** | 127 (100) | 135 (100) | 137 (100) |
| **Sex** |  |  |  |
| Male | 57 (44.9) | 62 (45.9) | 63 (46.0) |
| Female | 59 (46.4) | 61 (45.2) | 62 (45.3) |
| Missing data | 11 (8.7) | 12 (8.9) | 12 (8.8) |
| **Age at diagnosis** |  |  |  |
| ≤70 | 93 (73.2) | 99 (73.3) | 101 (73.7) |
| >70 | 31 (24.4) | 32 (23.7) | 32 (23.4) |
| Missing data | 3 (2.4) | 4 (3.0) | 4 (2.9) |
| **Smoking History** |  |  |  |
| Current + ex smoker | 87 (68.5) | 92 (68.1) | 94 (68.6) |
| Never smoker | 17 (13.4) | 18 (13.3) | 18 (13.1) |
| Missing data | 23 (18.1) | 25 (18.5) | 25 (18.2) |
| **TNM-stage at diagnosis** |  |  |  |
| IA | 50 (39.3) | 52 (38.5) | 53 (38.7) |
| IB | 13 (10.2) | 13 (9.6) | 13 (9.5) |
| IIA | 16 (12.6) | 16 (11.9) | 16 (11.7) |
| IIB | 21 (16.5) | 22 (16.3) | 22 (16.1) |
| IIIA | 17 (13.4) | 20 (14.8) | 21 (15.3) |
| IIIB | 0 (0.0) | 1 (0.7) | 1 (0.7) |
| IV | 1 (0.8) | 1 (0.7) | 1 (0.7) |
| Missing data | 9 (7.1) | 10 (7.4) | 10 (7.3) |
| **Histology** |  |  |  |
| Adenocarcinoma | 110 (86.6) | 117 (86.7) | 119 (89.5) |
| Squamous cell carcinoma | 3 (2.4) | 3 (2.2) | 3 (2.2) |
| NOS | 3 (2.4) | 3 (2.2) | 3 (2.2) |
| Missing data | 11 (8.7) | 12 (8.9) | 12 (8.8) |
| **WHO performance status** |  |  |  |
| 0 | n/a | n/a | n/a |
| 1 | n/a | n/a | n/a |
| 2 | n/a | n/a | n/a |
| 3 | n/a | n/a | n/a |
| 4 | n/a | n/a | n/a |
| **Mean follow-up (months)** | 48.6 | 49.9 | 51.8 |

FISH=fluorescence in situ hybridization

NOS=not otherwise specified

n/a=not applicable
